# Navigated Placement of Two Odontoid Screws Using the O-Arm Navigation System: A Technical Case Report

**DOI:** 10.7759/cureus.10724

**Published:** 2020-09-29

**Authors:** Clara K Starkweather, Ramin Morshed, Caleb Rutledge, Phiroz Tarapore

**Affiliations:** 1 Neurological Surgery, University of California San Francisco, San Francisco, USA; 2 Neurological Surgery, San Francisco General Hospital, San Francisco, USA; 3 Neurological Surgery, San Francisco Veteran's Hospital, San Francisco, USA

**Keywords:** navigated screw placement, o-arm, odontoid screw

## Abstract

Odontoid fractures are common cervical spine fractures and lead to atlantoaxial instability depending on their type. Fractures through the base of the odontoid neck are considered for surgery. While the management of these fractures is controversial and may include external immobilization or posterior fusion, an odontoid screw offers the advantages of directly crossing the fracture site while preserving motion at C1-2. Although intraoperative navigation is routinely utilized in spine surgery, there are few reports of navigated anterior odontoid screw placement. In this report, we describe the safe and accurate placement of two anterior odontoid screws using the O-arm navigation system in an octogenarian with a type II odontoid fracture. Details of the technical approach are also provided. The follow-up imaging at three months confirmed the healing of the fracture. Intraoperative navigation using the O-arm system allows for safe and accurate placement of two odontoid screws.

## Introduction

Odontoid fractures are common cervical spine fractures, particularly among the elderly [1,2]. These fractures are classified according to the Anderson and D’Alonzo classification [3]. Type II fractures involving the base of the odontoid neck are the most common type and are considered unstable, usually requiring prolonged external immobilization or surgical fixation [1]. Fixation can be achieved via both anterior and posterior approaches, including anterior odontoid screw fixation [4]. Unlike a posterior C1-2 Harms fusion, an odontoid screw directly crosses the fracture site and preserves motion at C1-2. Accurate screw placement is essential to ensure adequate fracture reduction and prevent neurological complications, and biplanar fluoroscopy with two C-arms is usually required.

Navigated screw placement increases the accuracy of screw placement in spine surgery [5,6]. However, there are few reports of navigated odontoid screw placement. Herein we report the navigated placement of two anterior odontoid screws using the O-arm navigation system, which is a portable imaging device that encircles the patient and works similar to a CT scanner to generate three-dimensional images intraoperatively. In this report, the O-arm navigation was used in an octogenarian with a type II odontoid fracture.

## Case presentation

**Figure 1:**
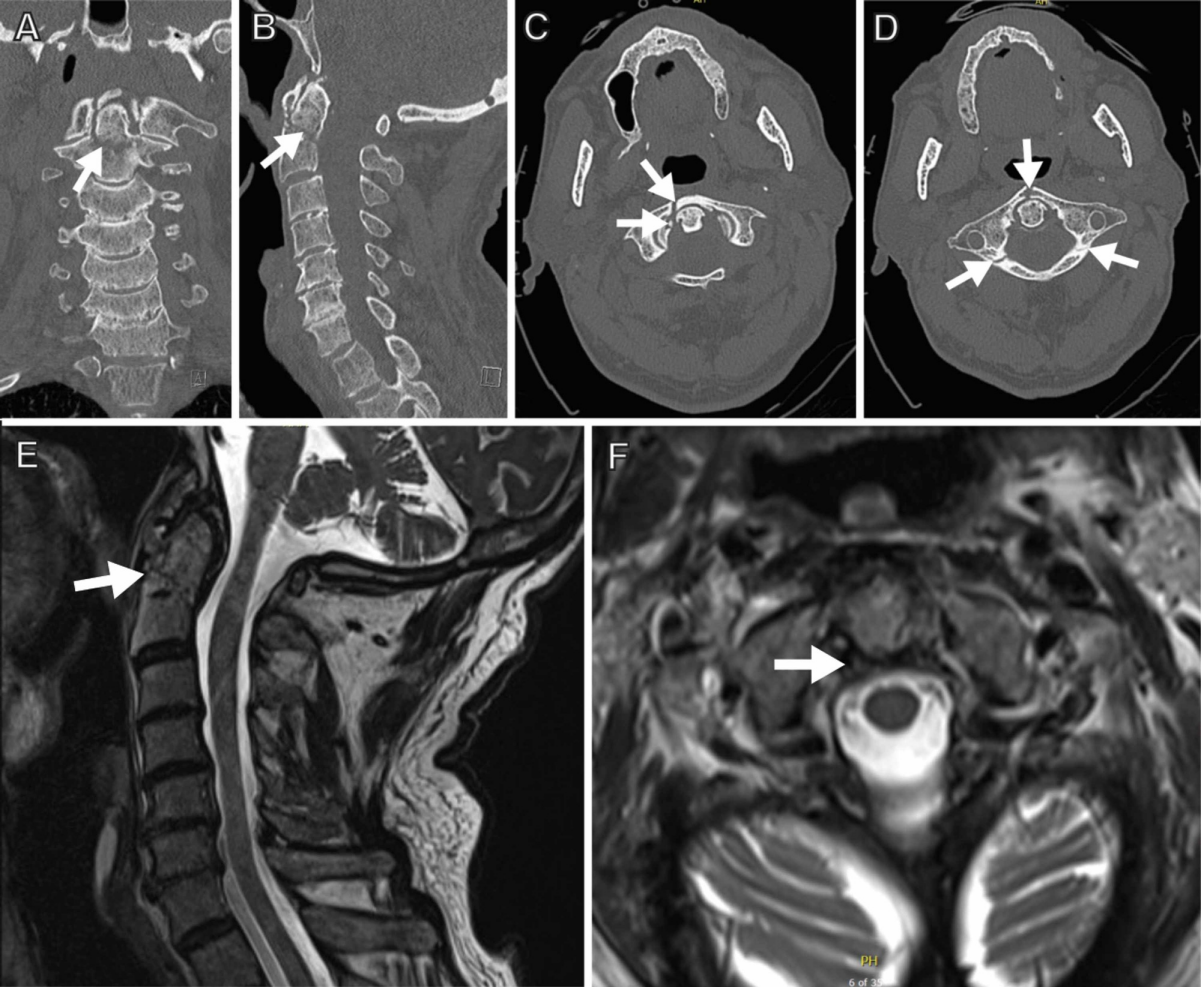
Preoperative images (A, B) coronal (A) and sagittal (B) CT views of the cervical spine demonstrate a type II odontoid fracture. (C, D) axial views demonstrate a chronic-appearing Jefferson fracture (chronic Jefferson fracture indicated by arrows). (E, F) T2-weighted MRI sagittal (E) and axial (F) images demonstrate the odontoid fracture (arrow in E) and an intact transverse ligament (arrow in F) CT: computed tomography; MRI: magnetic resonance imaging

An 86-year-old male presented after a motor vehicle accident with a well-corticated, chronic-appearing Jefferson fracture, and an acute type II odontoid fracture with fracture geometry suitable for odontoid screw reduction (Figure 1).

**Figure 2:**
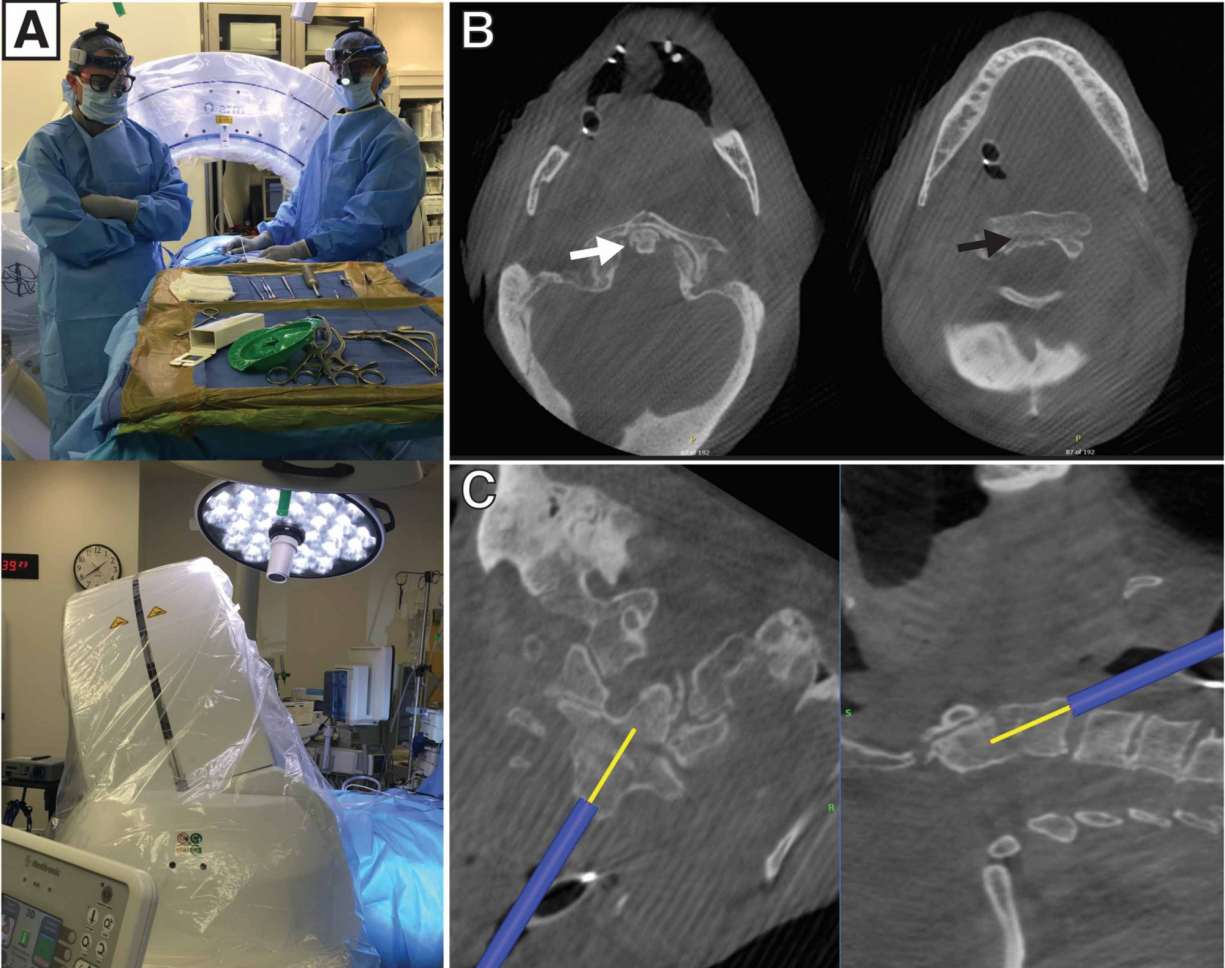
An intraoperative CT provides a real-time view of the fracture and spinal alignment after the patient positioning. The CT is then used to plan and navigate screw placement (A) O-arm set-up. (B) axial images demonstrating intraoperative visualization of the tip of the dens and C2 body, indicated by white and black arrows respectively, prior to screw placement. (C) visualization of screw trajectory CT: computed tomography

He was brought to the operating room and placed in a supine position on a regular bed. A Mayfield skull clamp was applied, and a large wad of cotton was placed in the mouth. The Medtronic arm and frame (Medtronic, Dublin, Ireland) were attached to the Mayfield clamp. The O-arm was used to obtain anteroposterior (AP) and lateral X-rays. After positioning, the patient was then prepped and draped in a sterile fashion (set-up displayed in Figure 2A), and the anterior surface of the vertebral bodies was exposed using a transverse skin incision at the C5-6 level. A combination of Metzenbaum scissors, bipolar cautery, and blunt dissection was used to expose the spine, similar to an anterior cervical discectomy and fusion approach. An Apfelbaum retractor system (Aesculap, Center Valley, PA) was used to retract soft tissue. AP and lateral X-rays were obtained to confirm the appropriate level, and a full O-arm spin was obtained (Figure 2B). Part of the C2-3 disk was removed to avoid anterior placement of the screw and compromise of the anterior cortex of C2. A handheld Stealth probe, which allows intraoperative navigation based on imaging, was then registered and used to determine an appropriate starting point and trajectory for the first odontoid screw (Figure 2C).

**Figure 3:**
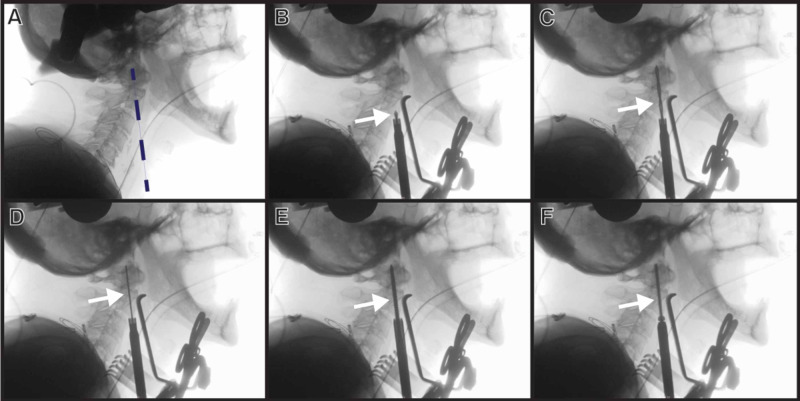
Planned trajectory of odontoid screw placement X-rays from the O-arm are used to (A) confirm planned trajectory (dotted line), (B) confirm drill position (drill tip indicated with the arrow), (C, D) visualize tapping (tap indicated with the arrow), and (E, F) visualize screw placement (screw indicated with the arrow). This eliminates the need for biplanar fluoroscopy and two C-arms

Fluoroscopy images of the following steps are displayed in Figure 3.

**Figure 4:**
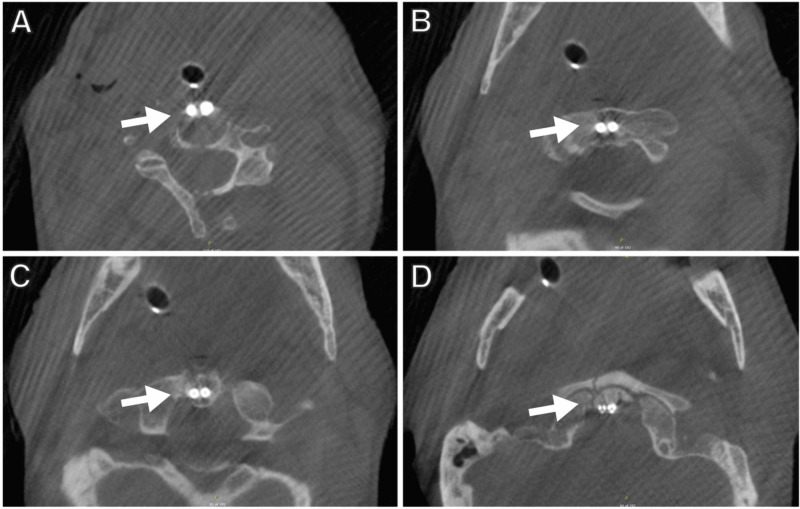
Final intraoperative images The final CT spine confirms placement of the odontoid screws (indicated with arrows) from (A) the anterior endplate of C2, traversing the (B) C2 body and (C) base of the odontoid process, and (D) penetrating the cortex of the odontoid tip CT: computed tomography

A pneumatic drill was used to create a pilot hole in the inferior aspect of C2. A drill guide registered to the Stealth system was then placed in the pilot hole, and a drill also registered to the Stealth system was used to drill through the C2 body across the fracture line toward the odontoid tip. AP and lateral X-rays were obtained to confirm the screw trajectory. The trajectory was tapped over a K-wire. After tapping, a screw was inserted under AP and lateral fluoroscopic guidance. The screw length was obtained from measurements from the O-arm-generated CT. A second trajectory was then planned and an additional odontoid screw was placed. A final O-arm spin was obtained to confirm the placement of the two screws and adequate reduction of the fracture (Figure 4).

**Figure 5:**
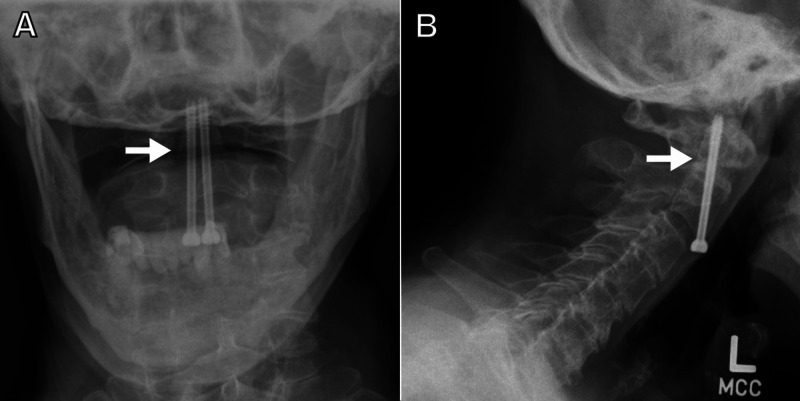
Three-month follow-up X-rays confirm healing of the fracture (A) AP and (B) lateral views of the cervical spine demonstrating union across former fracture site (arrows) AP: anteroposterior

The patient recovered well and was discharged in good condition in a hard cervical collar. The X-rays obtained at the last follow-up demonstrated adequate fusion (Figure 5).

## Discussion

Intraoperative navigation increases the accuracy of screw placement in spine surgery [7-9]. For example, Rajasekaran et al. demonstrated a significantly decreased rate of pedicle breach in thoracic pedicle screw placement when compared to non-navigated screw placement [9]. However, there are few reports of anterior odontoid screw placement using intraoperative navigation either with the O-arm or Iso-C systems [10-19]. Pisapia et al. recently compared outcomes for anterior odontoid screw fixation between navigated and non-navigated cases using the O-arm system. No malpositioned screws or neurovascular injury were reported although one patient in the navigated group had screw loosening and required posterior occipitocervical fusion [15]. Keskin et al. previously reported outcomes in 31 patients undergoing navigated anterior odontoid screw fixation using the Iso-C system. There were no malpositioned screws or neurovascular injury, although one patient required revision surgery due to non-union [13].

There are several advantages to using intraoperative navigation. An intraoperative CT provides a real-time view of the fracture and spinal alignment after the patient has been positioned. The CT is then used to plan and navigate the screw placement. In this case, we planned the placement of two odontoid screws to promote fusion, which is not feasible with standard biplanar fluoroscopy. Additionally, the built-in ability of the O-arm to obtain AP and lateral X-rays eliminates the need for biplanar fluoroscopy and two C-arms. Finally, the ability to obtain a final intraoperative CT to confirm screw placement and fracture reduction allows confirmatory imaging before the patient leaves the operating room. The limitations include its size, expense, and radiation exposure to providers (although the latter can be avoided to some extent).

In this report, we demonstrated that the navigated placement of two odontoid screws can be safe and feasible in an octogenarian patient. As geriatric patients are at an increased risk for insufficient bony healing and non-union [20], we hypothesized that this approach would optimize fracture reduction and provide additional stability across the fracture, thereby increasing the likelihood of union.

## Conclusions

The placement of anterior odontoid screws using the O-arm navigation system is technically feasible and safe. The O-arm provides real-time intraoperative anatomical visualization, including the fracture site and spinal alignment. In addition, the O-arm optimizes fracture reduction and screw placement. These factors allow for the placement of an additional screw, which may increase the likelihood of the union.
